# Kinetic Titration Series with Biolayer Interferometry

**DOI:** 10.1371/journal.pone.0106882

**Published:** 2014-09-17

**Authors:** Daniel Frenzel, Dieter Willbold

**Affiliations:** 1 Forschungszentrum Jülich, ICS-6 Structural Biochemistry, Jülich, Germany; 2 Heinrich-Heine-Universität Düsseldorf, Institut für Physikalische Biologie, Düsseldorf, Germany; University of Houston, United States of America

## Abstract

Biolayer interferometry is a method to analyze protein interactions in real-time. In this study, we illustrate the usefulness to quantitatively analyze high affinity protein ligand interactions employing a kinetic titration series for characterizing the interactions between two pairs of interaction patterns, in particular immunoglobulin G and protein G B1 as well as scFv IC16 and amyloid beta (1–42). Kinetic titration series are commonly used in surface plasmon resonance and involve sequential injections of analyte over a desired concentration range on a single ligand coated sensor chip without waiting for complete dissociation between the injections. We show that applying this method to biolayer interferometry is straightforward and i) circumvents problems in data evaluation caused by unavoidable sensor differences, ii) saves resources and iii) increases throughput if screening a multitude of different analyte/ligand combinations.

## Introduction

Surface plasmon resonance (SPR) is widely used to study analyte/ligand interactions in real-time [1]. For SPR analysis, one interactant (“ligand”) is immobilized onto the sensor surface while the other interactant (“analyte”) is passed over this surface by continuous flow. The standard assay requires complete removal of the analyte (“regeneration”) between each measurement cycle to avoid any residual analyte molecules blocking potential binding sites on the surface. This procedure is termed “multi-cycle kinetics” [2], because it consists of several cycles of alternating analyte injections and surface regeneration steps. The regeneration process often requires conditions that can inactivate the immobilized ligand irreversibly [3]. To avoid such potentially detrimental surface regeneration steps, a technique has been developed that allows several concentrations to be applied within a single cycle without the requirement of complete surface regeneration steps following each injection. These so called “kinetic titration series” or “single cycle kinetics” were found to be virtually as precise as classical “multi cycle kinetics” [4] and can be used as an alternative option if regeneration is not practical [5], [6]. Later kinetic titration series were adopted by GE with minor modifications.and renamed as “single-cycle kinetics” (Biacore manual).

More recently, biolayer interferometry (BLI) has become an alternative method to SPR. One advantage of BLI is that the number of sensors can be scaled up easily without making the system more error-prone or complex. Theoretically, there is no need to regenerate single sensors, because duplicates of the surface can be easily created by immobilizing an equal amount of ligand on additional sensors. However this approach has some disadvantages. It is not possible to achieve identical ligand coatings of multiple sensors. Such an approach also increases sensor consumption and it is not guaranteed that each sensor has an equal performance in later measurements.

We strove to overcome these disadvantages of BLI as compared with SPR by exploring whether application of a kinetic titration series in BLI experiments is feasible and accurate.

## Materials and Methods

### Preparation of protein G B1 (GB1) and scFv IC16

Purification of GB1 was done by standard Immunoglobulin G (IgG) affinity purification after expression in *E. coli* with pGEV2-GB1 [7] (see SI: “Preparation of protein G B1”) for details. Purification of scFv IC16 was done as described in Frenzel *et al.* [5].

### Immobilization of ligands via amine coupling on (AR2G) biosensors

The 40 µM GB1 solution in PBS was diluted in 10 mM sodium acetate buffer pH 4.0 to a final concentration of 20 µM (binding buffer). The sample sensors were pre-incubated in _dd_H_2_O for 10 min, activated in a 1∶1 mixture of 0.1 M N-Hydroxysuccinimide (NHS)/0.4 M 1-Ethyl-3-(3-dimethylaminopropyl)-carbodiimide (EDC) for 800 s and incubated in binding buffer for 900 s. The reference sensors were activated in the same way, but not treated with binding buffer. All sensors were blocked with 1 M ethanolamine for 180 s and stored in _dd_H_2_O before further usage. All steps were performed at 20°C with an agitation speed of 1000 rpm. For method definition and execution, the Data Acquisition software 7.1.0.92 from ForteBio was used.

### Immobilization of C-terminally biotinylated Aß(1–42) via amine coupling on Super SA-biosensors

C-terminally biotinylated Aβ(1–42) (EUROGENTEC) was dissolved in 100% HFIP and incubated at RT overnight. The stock solution was divided in 26.5 µg aliquots. HFIP was removed by evaporation in a Concentrator 5301 (Eppendorf). Aβ was freshly solubilized in 550 µl sodium phosphate buffer pH 7.4 (10 mM; yielding a 10 µM Aβ solution). To separate the monomers from bigger particles, they were subjected to a density gradient centrifugation (DGC) as described in Frenzel *et al*. [5]. After centrifugation, fraction one (140 µl) was used for immobilization of Aß(1–42) monomers via standard streptavidin-biotin-coupling procedure with Super SA-sensors (ForteBio). It was planned to immobilize 0.15 nm, 0.25 nm and 0.75 nm of ligand on eight sensors respectively. Further eight sensors were used as reference and remained in phosphate buffer. The finally achieved layer thickness of all sensors is summarized in Tab. 1.

**Table 1:** Final amount of immobilized protein on AR2G sensors.

Sensor:		1	2	3	4	5	6	7	8	Ø/SD
**A)**	**GB1[nm]:**	1.55	1.43	1.19	1.09	0.99	1.29	1.16	–	1.24/0.20
**B)**	**Aβ(1–42) [nm]:**	0.16	0.15	0.15	0.16	0.14	0.11	0.11	0.10	0.13/0.02
**C)**	**Aβ(1–42) [nm]:**	0.42	0.44	0.42	0.43	0.41	0.38	0.43	0.39	0.41/0.02
**D)**	**Aβ(1–42) [nm]:**	1.01	1.02	0.99	1.07	1.01	0.97	1.06	0.92	1.01/0.05

The value of the response is a measure of the amount of protein on the sensors. A) 1.25 nm, B) 0.15 nm, C) 0.25 nm and D) 0.75 nm have been defined as target for ligand immobilization. Ø and SD: Mean response and the corresponding standard deviation after immobilization.

### Parallel sensor kinetics of GB1 with biolayer interferometry

Kinetic titration series were performed in the interaction buffer (PBS with 0.05% Polysorbat 20). 5 mg/ml IgG (ID: ABIN376828; Antibodies-Online) was diluted in interaction buffer to 0.5 µM and further diluted four times with a dilution factor of two. To measure the interaction between IgG and GB1, the association and dissociation times were 360 and 600 s, respectively, for every analyte concentration. In total, five sensors were used to measure five different analyte concentrations in parallel, while one sensor was used to measure the buffer reference. Additional six sensors were used as sensor reference. All steps were performed at 25°C with an agitation speed of 1000 rpm. Sensorgrams were measured on an Octet Red96 (ForteBio) and double referenced against the buffer reference signal and the reference sensor signals using the Data Analysis software 7.1.0.36 (ForteBio). The double referenced sensorgrams were exported into the BiaEvaluation 4.1 compatible “csv”-format by a python script (see SI: “Scripts”). The sensorgrams obtained with the concentrations: 0.5, 0.25, 0.125, 0.0625 and 0.03125 µM were fitted with the BiaEvaluation software 4.1 from Biacore using a 1∶1 binding model that included an RI-term.

### Parallel sensor kinetics of scFv IC16 with biolayer interferometry

Kinetic titration series were performed in the interaction buffer (PBS with 0.5% Polysorbat 20, 0.1% BSA and 10% NSB reducer from GE Healthcare). 2.4 µM scFv IC16 was diluted four times with a dilution factor of two. To measure the interaction between Aß(1–42) and scFv IC16, the association and dissociation times were 270 and 90 s, respectively, for every analyte concentration. Further steps are comparable with “Parallel sensor kinetics of GB1 with biolayer interferometry” (Data Analysis software: 8.0.0.35). The sensorgrams with the concentrations: 0.24, 0.12, 0.06, 0.03 and 0.015 µM were fitted with the BiaEvaluation software 4.1 from Biacore using a 1∶1 binding model without RI-term.

### Kinetic titration series of GB1 with biolayer interferometry

To measure the affinity between IgG and GB1, the association and dissociation phases were recorded for 360 and 240 s, respectively, for every analyte concentration (same concentrations as described in: ”Parallel sensor kinetics of GB1 with biolayer interferometry”). Four sensors recorded the kinetic titration series, whereas one sensor recorded the buffer reference signal. Additional five sensors were used as sensor reference. All steps were performed at 25°C with an agitation speed of 1000 rpm. The sensorgrams were double referenced against the buffer reference signal and the empty sensors by the Data Analysis software 7.1.0.36 (ForteBio). The double referenced signals of each association and dissociation phase were combined and exported into a BiaEvaluation 4.1 compatible “csv”-format using a python script (SI: “Scripts”). The sensorgrams were fitted with the BiaEvaluation software 4.1 from Biacore with a 1∶1 kinetic titration series model that included an RI-term [4].

### Kinetic titration series of scFv IC16 with biolayer interferometry

To measure the affinity between IgG and GB1, the association and dissociation phases were recorded for 270 and 90 s, respectively, for every analyte concentration (same concentrations as described in “Parallel sensor kinetics of scFv IC16 with biolayer interferometry”). Five sensors recorded the kinetic titration series, whereas one sensor recorded the buffer reference signal and six sensors were used as sensor reference. The other steps are comparable with section “Kinetic titration series of GB1 with biolayer interferometry” (Data Analysis software: 8.0.0.35). The sensorgrams with the concentrations: 0.24, 0.12, 0.06, 0.03 and 0.015 µM were fitted with the BiaEvaluation software 4.1 from Biacore with a 1∶1 kinetic titration series model without RI-term [4].

## Results

### Ligand immobilization

In order to compare the practicality and efficiency between multi cycle kinetics and kinetic titration series using BLI, we used the well-studied interaction of GB1 with IgG. The reported dissociation coefficients (K_D_) of GB1 to the constant (Fc) region of IgGs are ∼0.1 µM (human IgG) and ∼0.77 µM (rabbit IgG) [8], [9]. The interaction of the scFv IC16 with Aβ(1–42) was used as an additional example system. ScFvs show in comparison to IgGs no avidity. The scFv IC16 is directed against the N-terminus of Aβ(1–42) and with SPR, a reliable K_D_ value of 0.76 µM for C-terminally biotinylated Aβ(1–42) monomers was already estimated [5].

GB1 was immobilized onto AR2G sensors via amine coupling. We found that at the end of the multiple immobilization procedures the amount of immobilized protein differed for each sensor (Tab. 1). The mean and standard deviation was 1.24 nm and 0.20 nm. Thus, homogeneous immobilization of protein to the surface was not possible, because the on-rates of the sensors seem to deviate from each other. We estimated fewer deviations with Streptavidin-Biotin coupling on “Super SA” sensor tips, especially at higher layer thicknesses. The sensors with 1.01 nm ligand had a standard deviation of 0.05 nm, which corresponds to 4.9%, based on the mean (Tab. 1A). However, with declining amount of ligand, the ratio of standard deviation to immobilized ligand grew (see Tab. 1B: 4.8% for 0.41 nm, SD: 0.02 nm and Tab. 1C: 15.4% for 0.13 nm, SD: 0.02 nm).

### Binding kinetics with parallel sensor kinetics

Parallel sensor kinetics is thought to be more precise than a kinetic titration series, because no secondary processes (like dissociation of previously bound analyte from the surface) impair the measurements. However, other effects like inhomogeneous coating and differences in sensitivity of single sensors are expected to compromise these precision advantages.

**Figure 1:**
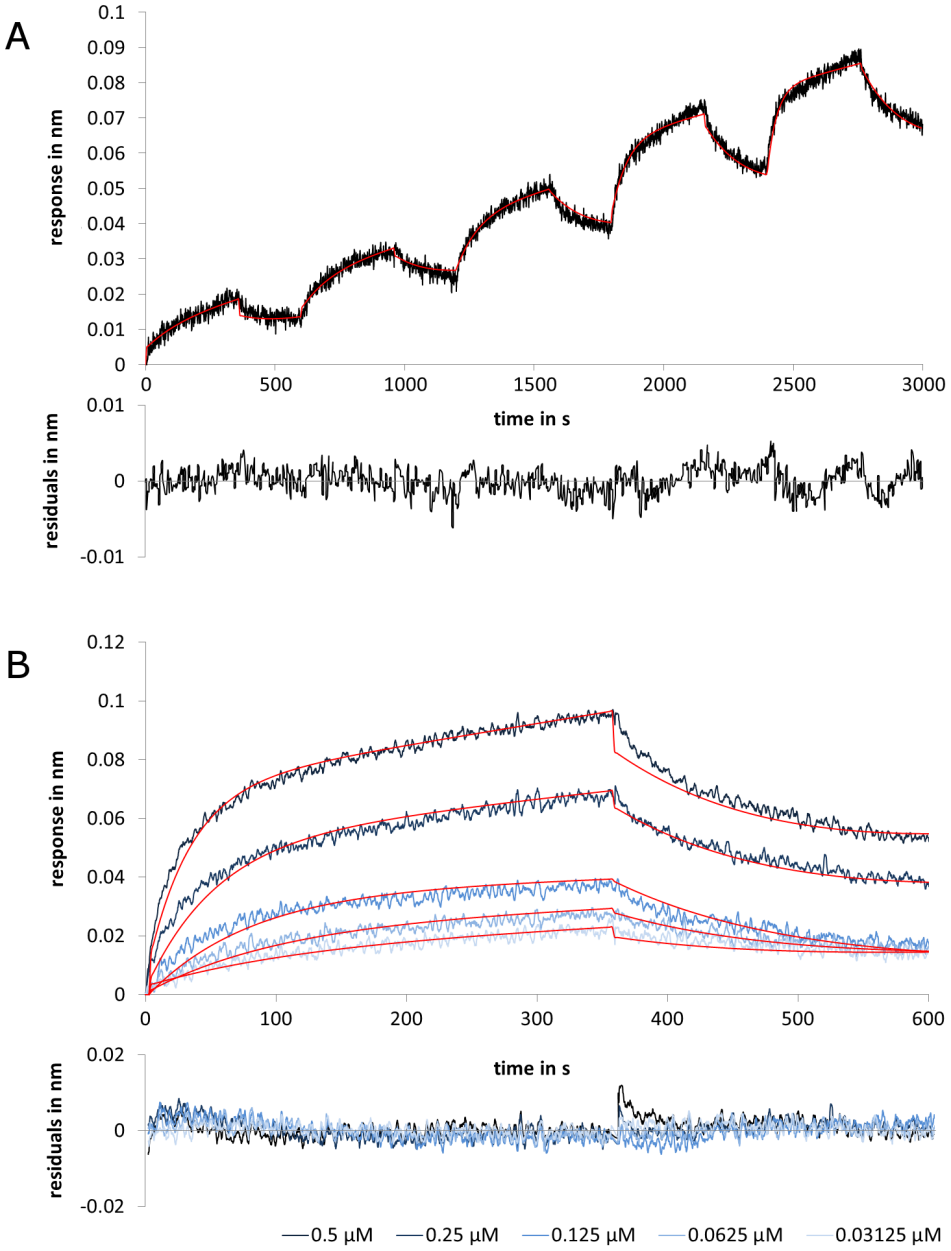
Evaluation of (A) kinetic titration series and (B) parallel sensor kinetics with rabbit IgG binding to GB1 in BLI. The sensorgrams show the interaction of IgG (analyte) with GB1 (ligand). Applied analyte concentrations were: 0.5, 0.25, 0.125, 0.0625 and 0.03125 µM. The fits are indicated by the red lines, whereas the sensorgrams are shown in black (A) and blue (B). The residuals of the fits are plotted below the respective sensorgram. All other experiments are shown in File S1.

**Figure 2:**
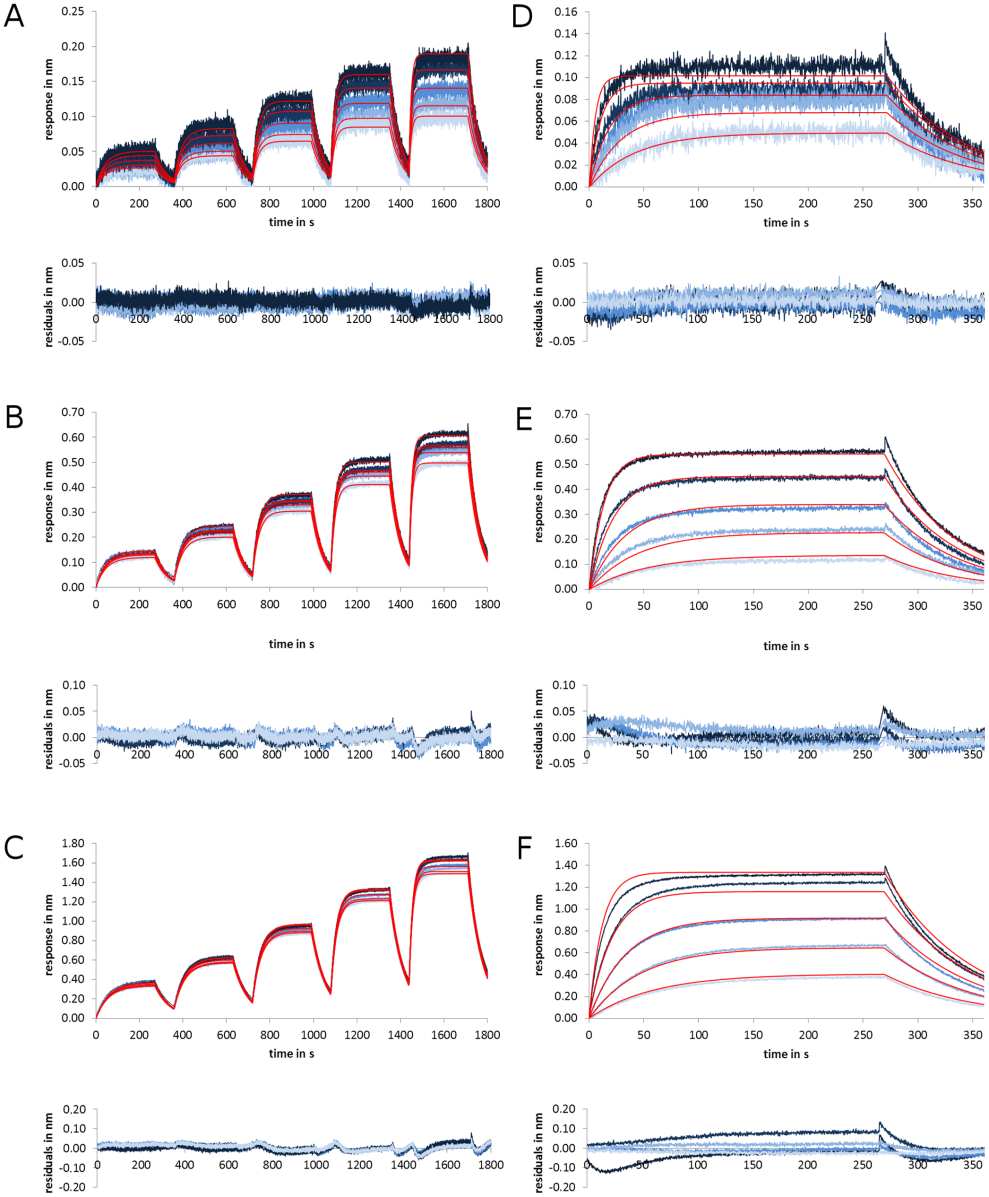
Comaprison of kinetic titration series (A–C) and parallel sensor kinetics (D–F) with scFv IC16 binding to Aβ(1–42) in BLI. The sensorgrams show the interaction of scFv IC16 (analyte) with C-terminally biotinylated Aß(1–42) (ligand). The amount of ligand was increased from 0.13 nm (A, D), 0.41 nm (B, E) and 1.01 nm (C, F). Applied analyte concentrations were: 2.4, 1.2, 0.6, 0.3 and 0.15 µM. The fits are indicated by the red lines, whereas the sensorgrams are shown in blue. Each kinetic titration series was reproduced five times. The residuals of the fits are plotted below the respective sensorgram.

To obtain data from parallel sensor kinetics, five sensor pairs were applied to record the signals received from applying five different analyte concentrations. One sensor pair was applied to record the buffer reference signal. Measurements with IgG and GB1 were fitted globally with RI-term (see: Fig. 1), whereas measurements with scFv IC16 and Aβ(1–42) were fitted without RI-term (see Fig. 2). The obtained *K*_D_s for GB1 were 0.16 µM and 0.25 µM. The χ^2^ values were 5.04×10^−6^ nm^2^ and 2.38×10^−6^ nm^2^. The term χ^2^ gives a measure for the accuracy of the fitting [10]. It represents the averaged, squared residual per data point. In our case, χ^2^ was below the squared sensor noise (∼0.008 nm), which is a quality indicator of a fit. With the scFv-system, we obtained 0.18 µM/χ^2^: 7.17×10^−5^ for 0.15 nm ligand, 0.59 µM/χ^2^: 2.25×10^−4^ for 0.41 nm ligand and 0.43 µM/χ^2^: 9.69×10^−4^ for 1.01 nm ligand (experiment was reproduced: data on request). Remarkably the K_D_s spread and just the best fit (see Fig. 2: E) is close to the expected affinity range [5].

### Binding kinetics with kinetic titration series

Sensorgrams from kinetic titration series were recorded for each amount of ligand from five (scFv IC16 and Aß1–42) and seven (IgG and GB1) sensor pairs (one sensor with ligand and one without) that were each subsequently applied to five different analyte concentrations within one titration series. Additionally one sensor pair was applied to record the buffer reference signals. Like for parallel sensor kinetics, measurements with GB1 were fitted with RI-term, whereas measurements with scFv IC16 were fitted without RI-term. Each sensor pair was fitted separately and each fit was used to calculate the mean values for the rate constants *k*_a_ and *k*_d_, as well as the dissociation constant *K*_D_. The estimated mean *K*_D_ of the interaction of IgG and GB1 was 0.16 µM (7 replicates, standard deviation: 0.08 µM) and the obtained mean χ^2^ was 4.92×10^−6^ nm^2^ (standard deviation: 2.15×10^−6^ nm^2^). By taking into account the standard deviation, the χ^2^ is virtually identical to the χ^2^ obtained for parallel sensor kinetics. Comparison of the on-rates (*k*_a_), off-rates (*k*_d_) and binding constants (*K*_D_) for IgG and GB1 showed that both methods give near identical values (Tab. 2). Both, the on-rates and the off-rates lie within the same range of 10^4^ 1/Ms and 10^−3^ 1/s respectively (Fig. 1).

**Table 2:** Comparison of the binding constants obtained by fitting with equivalent models.

Kinetic titration series					Parallel sensor kinetics				
	K_a_ [1/Ms)	k_d_ (1/s)	K_D_ (M)	?^2^		k_a_ (1/Ms)	k_d_ (1/s)	K_D_ (M)	?^2^
**IgG/GB1 Ø (n = 7)**	4.78E-03	3.49E+04	1.59E-07	4.92E-06	**IgG/GB1 #1**	7.02E-03	4.51E-04	1.56E-07	5.04E-06
					**IgG/GB1 #2**	2.28E+04	5.78E-03	2.53E-07	2.38E-06
**IC16/Aβ(1–42) @0.15 nm Ø (n = 5)**	3.36E+04	1.83E-02	5.43E-07	3.87E-05	**IC16/Aβ(1–42)** @0.15 ** nm**	7.24E+04	1.32E-02	1.83E-07	7.17E-05
**IC16/Aβ(1–42) @0.41 nm Ø (n = 5)**	2.74E+04	1.76E-02	6.42E-07	6.80E-05	**IC16/Aβ(1–42)** @0.41 ** nm**	2.55E+04	1.51E-02	5.94E-07	2.25E-04
**IC16/Aβ(1–42) @1.01 nm Ø (n = 5)**	2.03E+04	1.43E-02	7.08E-07	2.44E-04	**IC16/Aβ(1–42)** @1.01 ** nm**	2.95E+04	1.27E-02	4.31E-07	9.69E-04

Ø: mean, SD: standard deviation, *k*_d_: off rate in 1/s, *k*_a_: on rate in 1/Ms, *K*_D_: dissociation constant in M, #1/2: measurement one and two.

With the scFv-system we estimated the K_D_s: 0.54 µM/χ^2^: 3.87×10^−5^ for 0.15 nm ligand, 0.64 µM/χ^2^: 6.80×10^−5^ for 0.41 nm ligand and 0.71 µM/χ^2^: 2.44×10^−4^ for 1.01 nm ligand (experiment was reproduced with three sensor pairs: data on request). It is obvious, that for every amount of ligand, the χ^2^-term is at least several times smaller in comparison to parallel sensor kinetics (see Fig. 2: A/B/C vs Fig. 2: D/E/F) and all the estimated K_D_s (see Tab. 2 and Tab. S1 in File S1) are very close to the value estimated by SPR [5].

A known problem of using the χ^2^ rating is that this method is based on averaging. Local deviations can hardly be evaluated with χ^2^ alone. If one fit with a higher χ^2^ and one with a smaller χ^2^ are compared with each other, it is possible that the fit with the smaller χ^2^ has higher local deviations from the sensorgram. In contrast, the fit with the higher χ^2^ could proceed completely within the noise pattern. In this example, the fit with the higher χ^2^ could be the more accurate description of the sensorgram. In our case, the fit curves of the kinetic titration series are frequently within the range of the sensor noise, whereas the fit of the parallel sensor kinetics is outside the noise range at certain time points (Fig. 1A/Fig. S1 in File S1: t_360 s_, t_960 s_, t_1560 s_, t_2160 s_, Fig. 1B/Fig. S2 in File S1: t_0–60s_ and t_360–400 s_ and t_2760 s_; Fig. 2). This illustrates that the kinetic titration series can yield more reliable fits, because the affinity differences of single sensors are omitted since only one sensor pair is used per interaction study. This is most obvious if the sensorgrams are fitted without RI-term or local R_max_ as a linear correction mechanism (Fig. 2: A–F and Fig. S3 in File S1).

## Discussion

For our example system rabbit IgG and GB1, kinetic titration series and parallel sensor kinetics provided near identical results with regard to the on/off-rates as well as the *K*_D_ values and are in accord to published data. The mean χ^2^ values (considering all sensor data of either the kinetic titration series or parallel sensor kinetics) were nearly identical in both methods. The other example system, utilizing the interaction of scFv IC16 and Aβ(1–42) was fitted without RI-term. The result was showing a clear advantage of the kinetic titration series in respect to χ^2^-values the reliability of the estimated K_D_ at every immobilization level. However, it is not advisable to rate fits based only on the χ^2^. This is why we consider local deviations of the fits from the sensorgrams as another marker for the quality of the fit. With regard to this point, fits of parallel sensor kinetics have stronger local deviations from the sensorgrams for each recorded concentration, whereas the kinetic titration series yielded single fits with a lower degree of local deviations from the sensorgrams.

We have described possible approaches to design and evaluate a kinetic titration series with a 1∶1 binding model with and without RI-term using BLI. Implementing more complex binding models that deal with heterogeneous ligands or bivalent analytes [5] should be straightforward and allow more sophisticated analyses. We conclude that kinetic titration series for BLI are able to yield reliable fits that are at least as precise as parallel sensor kinetics. An additional advantage of the kinetic titration series is the potential enhancement of assay throughput and savings of resources by reduction of sensor consumption per ligand-analyte analysis, which is especially useful in environments like pharmaceutical industry were a high throughput is aimed.

## Supporting Information

File S1 **Supporting files. Figure S1, Repetitions of the kinetic titration series**. A–F) Measurements are indicated in black and the corresponding fit by a red line. Below each sensorgram is a plot of the respective fit residuals. **Figure S2, Repetition of the parallel sensor kinetics**. The sensorgrams are indicated by blue lines in different darkness and the corresponding fits by red lines. Below are the plots of the respective fit residuals given in the same blue as above. **Figure S3, Comparison of the fitting models without use of the RI term**. A) Fit of parallel sensor kinetics without RI and residual plots below. B) Fit of kinetic titration series without RI and the respective residual plot below. **Method S1, Preparation of protein G B1. Table S1,** **Comprehensive table of all evaluated fits**. F1A/B: Fitting results for the measurements illustrated in Fig. 1. S1A–S1F/S2/S3: fitting results for the measurements illustrated in Fig. S1, Fig. S2 and Fig. S3. k_a_: on-rate constant, k_d_: off-rate constant, K_D_: dissociation constant (k_d_/k_a_), RI_1_–RI_5_: baseline drift in nm, Χ^2^: chi^2^ in nm^2^. **Script S1, Example script (Python) for combining BLI raw data.** This example script illustrates how to combine the raw data (after export) from the ForteBio software to a unified single cycle kinetic for import by third party software. **Script S2, Residual calculation of kinetic titration series.** Script to calculate a residual table from the exported fits based on the measurements after data export in a straight forward way.
(DOCX)
