# Supplementary material for: Kinetic Titration Series with Biolayer Interferometry
Source: PLoS One. 2014 Sep 17;9(9):e106882. doi: 10.1371/journal.pone.0106882 (PMC4167697; doi:10.1371/journal.pone.0106882)
Supplement: File S1 — Supporting files. Figure S1, Repetitions of the kinetic titration series. A–F) Measurements are indicated in black and the corresponding fit by a red line. Below each sensorgram is a plot of the respective fit residuals. Figure S2, Repetition of the parallel sensor kinetics. The sensorgrams are indicated by blue lines in different darkness and the corresponding fits by red lines. Below are the plots of the respective fit residuals given in the same blue as above. Figure S3, Comparison of the fitting models without use of the RI term. A) Fit of parallel sensor kinetics without RI and residual plots below. B) Fit of kinetic titration series without RI and the respective residual plot below. Method S1, Preparation of protein G B1. Table S1, Comprehensive table of all evaluated fits. F1A/B: Fitting results for the measurements illustrated in Fig. 1. S1A–S1F/S2/S3: fitting results for the measurements illustrated in Fig. S1, Fig. S2 and Fig. S3. ka: on-rate constant, kd: off-rate constant, KD: dissociation constant (kd/ka), RI1–RI5: baseline drift in nm, Χ2: chi2 in nm2. Script S1, Example script (Python) for combining BLI raw data. This example script illustrates how to combine the raw data (after export) from the ForteBio software to a unified single cycle kinetic for import by third party software. Script S2, Residual calculation of kinetic titration series. Script to calculate a residual table from the exported fits based on the measurements after data export in a straight forward way. (DOCX) [file pone.0106882.s001.docx]

***Supplementary information***

# Kinetic titration series with biolayer interferometry

Daniel Frenzel^1^, Dieter Willbold^1,2*^

^1^Forschungszentrum Jülich, ICS-6 Structural Biochemistry, 52428 Jülich, Germany

^2^Heinrich-Heine-Universität Düsseldorf, Institut für Physikalische Biologie, 40225 Düsseldorf, Germany

^*^Address correspondence to: D.Willbold@fz-juelich.de

Methods:

Method S1: Preparation of protein G B1

Chemically competent *E. coli* BL21 DE3 pRARE2 cells were transformed with the expression vector pGEV2-GB1 [7]. A 0.5 l LB (5 g/l yeast extract, 10 g/l tryptone, 10 g/l NaCl, 2 g/l dextrose, 10 mM MgCl_2_, chloramphenicol and ampicillin, pH 7.4) expression culture was inoculated with a 50 ml overnight culture in LB (5 g/l yeast extract, 10 g/l tryptone, 10 g/l NaCl) culture (grown at 37 °C, 150 rpm) to a final OD_600_ of ~0.1. Cells were grown at 37 °C (150 rpm) to an OD_600_ of 0.6 until IPTG was added to a final concentration of 1 mM for induction of GB1 expression. Expression was continued under the same conditions for 6 h. Cells were harvested by centrifugation (30 min, 4 °C, 3,500 *g*), pellets washed with PBS (10 mM sodium phosphate buffer pH 7.4, 137 mM NaCl, 2.7 mM KCl) and resuspended in 20 ml lysis buffer I (50 mM Tris-HCl pH 8.0, 1 mM EDTA, 1 mg/ml lysozyme) supplemented with protease inhibitors (complete EDTA-free Protease Inhibitor Cocktail Tablets, Roche). For cell lysis, 20% Triton X-100 was added to a final concentration of 1%. MgCl_2_ was added to a final concentration of 20 mM together with 500 U DNAse I. After incubation at RT for ~15 minutes, the volume was adjusted to 50 ml with lysis buffer II (16.6 mM MgCl_2_, 16.6 mM CaCl_2_, 1% Triton X-100) followed by centrifugation for 30 min at 20,000 *g*. Purification was done with 5 ml Immunoglobulin G (IgG) agarose from Qiagen that was equilibrated with lysis buffer I and II. The supernatant was loaded onto the column by gravity flow, followed by washing with ten column volumes of PBS. GB1 was eluted with 50 mM glycine, pH 2.5. All fractions were analyzed by SDS-PAGE with Coomassie Brilliant BlueR-250 staining and GB1 containing fractions were pooled, dialyzed against PBS and concentrated to 40 µM with Vivaspin 20 columns from Sartorius Stedim (3000 MWCO PES).

Supplementary Figures:


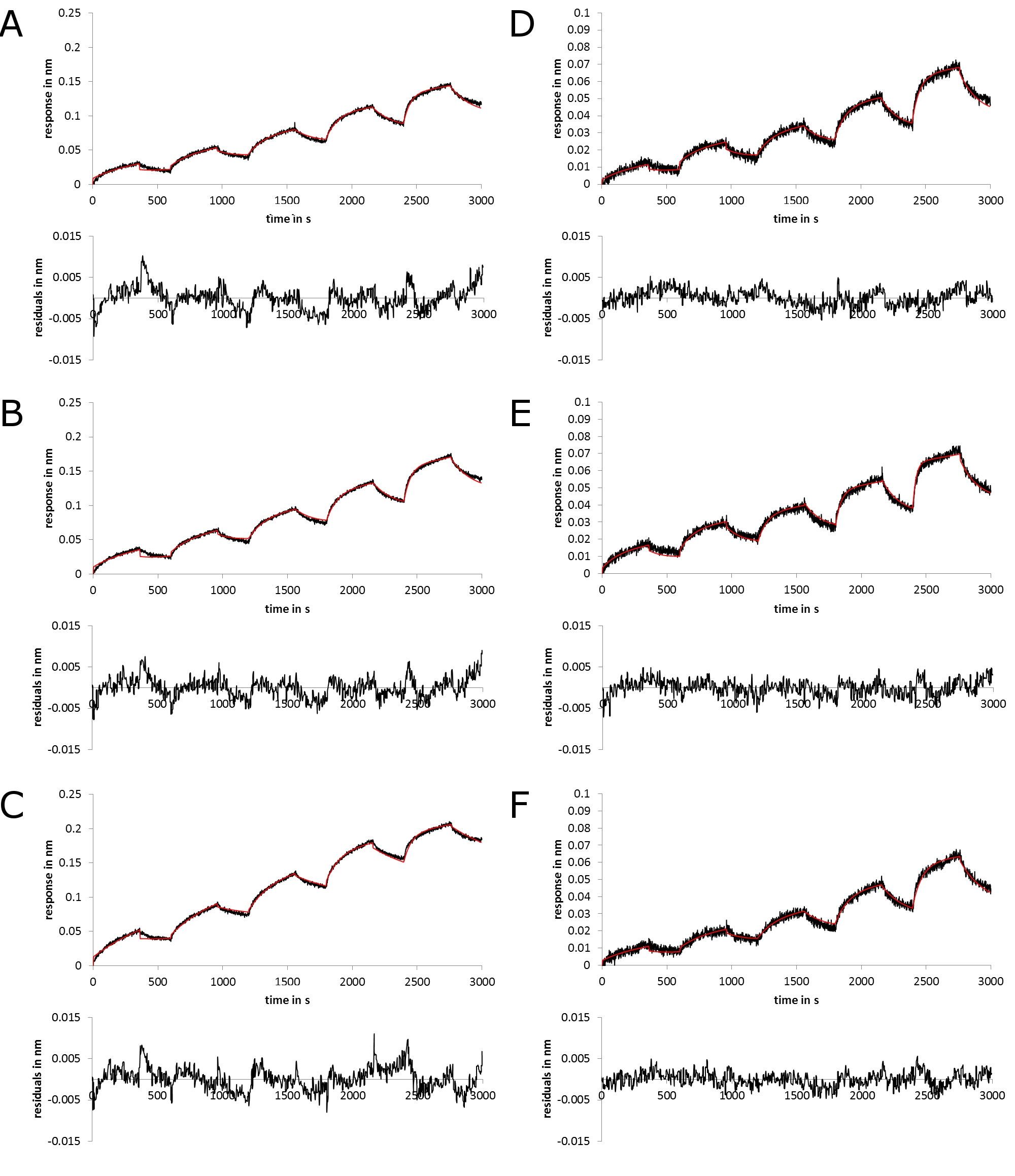


**Figure S1**: **Repetitions of the kinetic titration series**. A-F) Measurements are indicated in black and the corresponding fit by a red line. Below each sensorgram is a plot of the respective fit residuals.


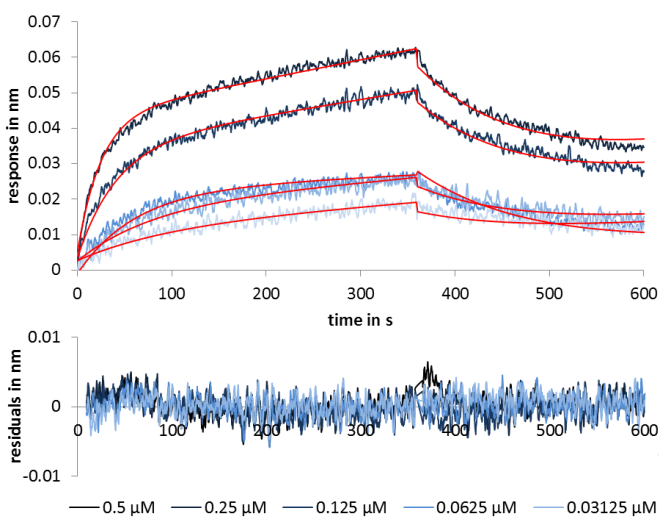


**Figure S2**: **Repetition of the parallel sensor kinetics**. The sensorgrams are indicated by blue lines in different darkness and the corresponding fits by red lines. Below are the plots of the respective fit residuals given in the same blue as above.


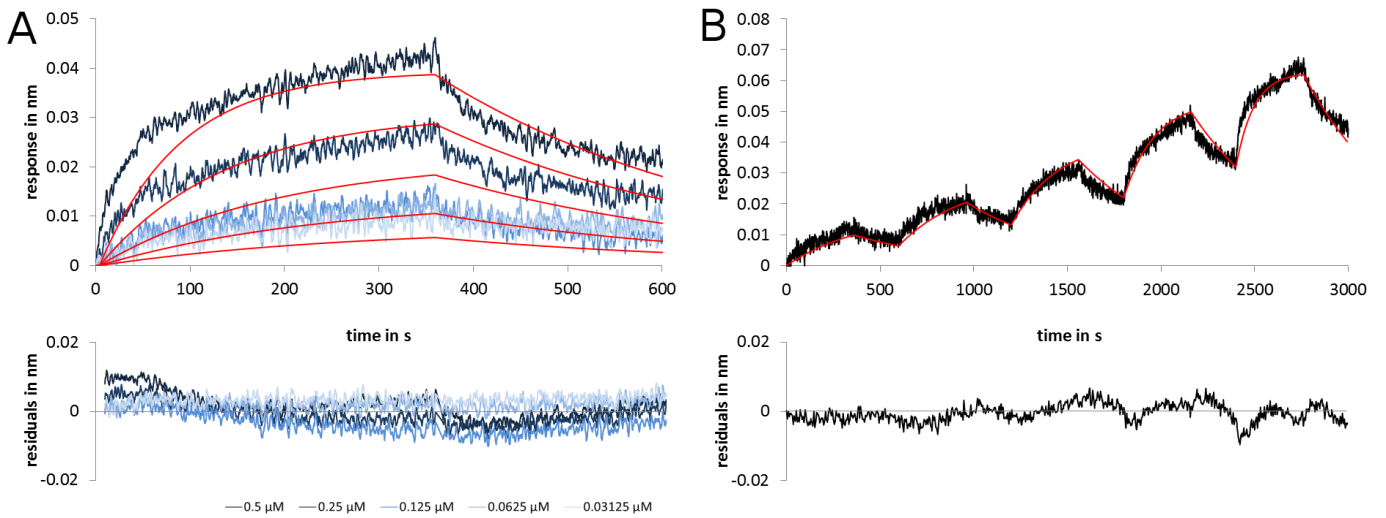


**Figure S3: Comparison of the fitting models without use of the RI term**. A) Fit of parallel sensor kinetics without RI and residual plots below. B) Fit of kinetic titration series without RI and the respective residual plot below.

Tables:

**Table S1**: **Comprehensive table of all evaluated fits**. F1A/B: Fitting results for the measurements illustrated in Fig. 1. S1A-S1F/S2/S3: fitting results for the measurements illustrated in Fig. S1, Fig. S2 and Fig. S3. k_a_: on-rate constant, k_d_: off-rate constant, K_D_: dissociation constant (k_d_/k_a_), RI_1_-RI_5_: baseline drift in nm, Χ²: chi² in nm².

|  | **k_a_ (1/Ms)** | **k_d_ (1/s)** | **Rmax (nm)** | **K_D_ (M)** | **RI_1_** | **RI_2_** | **RI_3_** | **RI_4_** | **RI_5_** | **Χ²** |
| --- | --- | --- | --- | --- | --- | --- | --- | --- | --- | --- |
| **F1A** | 4.43E+04 | 5.51E-03 | 0.038 | 1.24e-07 | 4.59E-03 | 2.08E-03 | 4.48E-04 | 3.45E-03 | 8.57E-04 | 2.87E-06 |
| **F1B** | 4.51E+04 | 7.02E-03 | 0.077 | 1.56e-07 | 3.23E-03 | 1.21E-03 | 2.45E-04 | 4.49E-03 | 1.16E-02 | 5.04E-06 |
| **F2A** | 3.36E+04 | 1.83E-02 | n.a. | 5.43E-07 | 0 | 0 | 0 | 0 | 0 | 3.87E-05 |
| **F2B** | 2.74E+04 | 1.76E-02 | n.a. | 6.42E-07 | 0 | 0 | 0 | 0 | 0 | 6.80E-05 |
| **F2C** | 2.03E+04 | 1.43E-02 | n.a. | 7.08E-07 | 0 | 0 | 0 | 0 | 0 | 2.44E-04 |
| **F2D** | 7.24E+04 | 1.32E-02 | 0.109 | 1.83E-07 | 0 | 0 | 0 | 0 | 0 | 7.17E-05 |
| **F2E** | 2.55E+04 | 1.51E-02 | 0.676 | 5.94E-07 | 0 | 0 | 0 | 0 | 0 | 2.25E-04 |
| **F2F** | 2.95E+04 | 1.27E-02 | 1.580 | 4.31E-07 | 0 | 0 | 0 | 0 | 0 | 9.69E-04 |
| **S1A** | 2.87E+04 | 4.76E-03 | 0.076 | 1.66e-07 | 8.55E-03 | 4.66E-03 | 3.92E-04 | 7.50E-04 | 1.24E-03 | 6.04E-06 |
| **S1B** | 2.97E+04 | 4.85E-03 | 0.087 | 1.64e-07 | 1.01E-02 | 5.63E-03 | 1.35E-04 | -4.08E-05 | 1.88E-03 | 7.55E-06 |
| **S1C** | 3.18E+04 | 1.60E-03 | 0.113 | 5.04e-08 | 1.26E-02 | 4.89E-03 | 3.03E-03 | 7.77E-03 | 2.42E-03 | 7.80E-06 |
| **S1D** | 6.85E+04 | 6.01E-03 | 0.035 | 8.77e-08 | 3.09E-03 | 3.35E-03 | -1.39E-03 | -8.12E-04 | 3.24E-03 | 3.59E-06 |
| **S1E** | 2.28E+04 | 6.06E-03 | 0.046 | 2.66e-07 | 2.56E-03 | 4.21E-03 | 3.68E-05 | 1.02E-03 | 2.85E-03 | 3.40E-06 |
| **S1F** | 1.84E+04 | 4.67E-03 | 0.048 | 2.54e-07 | 2.69E-03 | 2.28E-03 | 1.69E-04 | 9.26E-04 | 2.42E-03 | 3.18E-06 |
| **S2** | 2.28E+04 | 5.78E-03 | 0.037 | 2.53e-07 | 2.39E-03 | 1.67E-03 | 3.95E-04 | 3.61E-03 | 8.80E-03 | 2.38E-06 |
| **S3A** | 1.70E+04 | 3.17E-03 | 0.054 | 1.86e-07 | 0 | 0 | 0 | 0 | 0 | 1.42E-05 |
| **S3B** | 1.73E+04 | 1.85E-03 | 0.076 | 1.07e-07 | 0 | 0 | 0 | 0 | 0 | 6.95E-06 |

Scripts:

**Script S1: Example script (Python) for combining BLI raw data.** This example script illustrates how to combine the raw data (after export) from the ForteBio software to a unified single cycle kinetic for import by third party software.

import csv

import numpy

import math

import glob

import os

import sys

import random

from collections import defaultdict

def open_csv**(**filename**,** skiprows **=** 4**):**

columns **=** defaultdict**(**list**)** #we want a list to append each value in each column to

headers **=** **[]**

# Read the csv table

with open**(**filename**,** 'rb'**)** as f**:**

**for** line in xrange**(**skiprows**):**

f**.**readline**()**

reader **=** csv**.**DictReader**(**f**,** delimiter**=**'\t'**,** quotechar**=**'|'**)** #create a reader which represents rows in a dictionary form

counter **=** 0

**for** row in reader**:** #**this** will read a row as **{**column1**:** value1**,** column2**:** value2**,...}**

# Read in headers of the table

**if** **not** counter**:**

**for** **(**k**,** v**)** in row**.**items**():**

headers**.**append**(**k**)**

# Save the columns

**for** **(**k**,** v**)** in row**.**items**():** #go over each column name **and** value

columns**[**k**].**append**(**v**)** #append the value into the appropriate list based on column name k

counter **+=** 1

**return** headers**,** columns

# Count the number of datasets in this table

def nr_of_datasets**(**headers**):**

datasets **=** 0

**for** i in headers**:**

**if** "Data" **and** "Time" in i**:**

datasets **+=** 1

**return** datasets

def create_mat**(**columns**,** sets**):**

matrix **=** **[]**

**for** i in xrange**(**sets**):**

ncolT **=** "Time%d" **%** **(**i**+**1**)** # from 1 to **..**

ncolD **=** "Data%d" **%** **(**i**+**1**)** # from 1 to **..**

**if** len**(**columns**[**ncolT**])** **==** len**(**columns**[**ncolD**]):**

length **=** len**(**columns**[**ncolT**])**

x **=** **[]**

y **=** **[]**

# read the time

**for** j in columns**[**ncolT**]:**

j **=** j**.**replace**(**','**,** '.'**)**

fNum **=** round**(**float**(**j**),** 1**)**

x**.**append**(**fNum**)**

#x.append(fNum+0.1)

# read the values measured

**for** j in columns**[**ncolD**]:**

j **=** j**.**replace**(**','**,** '.'**)**

fNum **=** float**(**j**)**

y**.**append**(**fNum**)**

matrix**.**append**(**x**)**

matrix**.**append**(**y**)**

**else:**

print "Column sizes are not matching"

**return** matrix

def unify_dataset**(**matrix**,** sets**):**

uni_x **=** **[]**

uni_y **=** **[]**

dt **=** 0

dy **=** 0

# align y values at zero

dy **=** matrix**[**1**][**0**]**

# unify partial measurements

**for** i in xrange**(**sets**):**

cur_x **=** matrix**[**2*****i**]**

cur_y **=** matrix**[**2*****i**+**1**]**

**if** i **>** 0**:**

dt **+=** cur_x**[**len**(**cur_x**)-**1**]**

**for** j in xrange**(**len**(**cur_x**)):**

uni_x**.**append**(**cur_x**[**j**]** **+** dt**)**

**for** j in xrange**(**len**(**cur_y**)):**

uni_y**.**append**(**cur_y**[**j**]** **-** dy**)**

# Extent the end of the measurement

timer **=** uni_x**[-**1**]**

last **=** uni_y**[-**1**]**

drop_const **=** **(**uni_y**[-**10**]** **-** uni_y**[-**1**])** **/** **(**0.2 ***** 10**)**

matrix **=** **[]**

matrix**.**append**(**uni_x**)**

matrix**.**append**(**uni_y**)**

**return** matrix

def make_csv**(**filename**,** headers**,** matrix**):**

with open**(**filename**,** 'wb'**)** as csvfile**:**

spamwriter **=** csv**.**writer**(**csvfile**,** delimiter**=**'\t'**,** quotechar**=**'|'**,** quoting**=**csv**.**QUOTE_MINIMAL**)**

spamwriter**.**writerow**([**"time (s)"**,** "response (nm)"**])**

x **=** matrix**[**0**]**

y **=** matrix**[**1**]**

**for** i in xrange**(**len**(**x**)):**

xstr **=** "%.1f" **%** **(**x**[**i**])**

xstr **=** xstr**.**replace**(**'.'**,** ','**)**

ystr **=** "%.4f" **%** **(**y**[**i**])**

ystr **=** ystr**.**replace**(**'.'**,** ','**)**

spamwriter**.**writerow**([**xstr**,** ystr**])**

def batch_files**(**directory**):**

os**.**chdir**(**directory**)**

**for** file in glob**.**glob**(**"*.xls"**):**

headers**,** columns **=** open_csv**(**file**)**

datasets **=** nr_of_datasets**(**headers**)**

mat **=** create_mat**(**columns**,** datasets**)**

mat **=** unify_dataset**(**mat**,** datasets**)**

out_name **=** "sck_genera_" **+** file

out_name **=** out_name**.**replace**(**".xls"**,** ".csv"**)**

make_csv**(**out_name**,** headers**,** mat**)**

**if** len**(**sys**.**argv**)** **<** 2**:**

print 'No parameters.'

sys**.**exit**()**

**if** sys**.**argv**[**1**].**startswith**(**'--'**):**

option **=** sys**.**argv**[**1**][**2**:]**

# hole sys.argv[1], aber ohne die ersten beiden Zeichen

**if** option **==** 'help'**:**

print '''Syntax: script "filename"'''

**else:**

print 'unknown option.'

sys**.**exit**()**

**else:**

**for** directory in sys**.**argv**[**1**:]:**

print directory

batch_files**(**directory**)**

**Script S2: Residual calculation of kinetic titration series.** Script to calculate a residual table from the exported fits based on the measurements after data export in a straight forward way.

**import** csv

**import** numpy

**import** math

**import** glob

**import** os

**import** sys

**import** random

**from** collections **import** defaultdict

**def** open_csv**(**filename**,** skiprows **=** 0**):**

columns **=** defaultdict**(**list**)** #we want a list to append each value in each column to

headers **=** **[]**

# Read the csv table

**with** open**(**filename**,** 'rb'**)** **as** f**:**

**for** line **in** xrange**(**skiprows**):**

f**.**readline**()**

reader **=** csv**.**DictReader**(**f**,** delimiter**=**';'**,** quotechar**=**'|'**)** #create a reader which represents rows in a dictionary form

counter **=** 0

**for** row **in** reader**:** #this will read a row as {column1: value1, column2: value2,...}

# Read in headers of the table

**if** **not** counter**:**

**for** **(**k**,** v**)** **in** row**.**items**():**

headers**.**append**(**k**)**

# Save the columns

**for** **(**k**,** v**)** **in** row**.**items**():** #go over each column name and value

columns**[**k**].**append**(**v**)** #append the value into the appropriate list based on column name k

counter **+=** 1

**return** headers**,** columns

# Count the number of datasets in this table

**def** nr_of_datasets**(**headers**):**

datasets **=** 0

**for** i **in** headers**:**

**if** "Data" **and** "Time" **in** i**:**

datasets **+=** 1

**return** datasets

**def** create_mat**(**columns**,** sets**):**

matrix **=** **[]**

**for** i **in** xrange**(**sets**):**

ncolT **=** "Time%d" **%** **(**i**+**1**)** # from 1 to ..

ncolD **=** "Data%d" **%** **(**i**+**1**)** # from 1 to ..

**if** len**(**columns**[**ncolT**])** **==** len**(**columns**[**ncolD**]):**

length **=** len**(**columns**[**ncolT**])**

x **=** **[]**

y **=** **[]**

# read the time

**for** j **in** columns**[**ncolT**]:**

j **=** j**.**replace**(**','**,** '.'**)**

**if** j **!=** ""**:**

fNum **=** round**(**float**(**j**),** 1**)**

x**.**append**(**fNum**)**

x**.**append**(**fNum **+** 0.1**)**

# read the values measured with 0.2s to 0.1s interpolation

last **=** 0

**for** j **in** columns**[**ncolD**]:**

**if** j **!=** ""**:**

j **=** j**.**replace**(**','**,** '.'**)**

fNum **=** float**(**j**)**

y**.**append**(**fNum**)**

y**.**append**((**fNum **+** last**)** **/** 2**)**

last **=** fNum

matrix**.**append**(**x**)**

matrix**.**append**(**y**)**

**else:**

**print** "Column sizes are not matching"

**return** matrix

**def** calc_diff**(**matrix**):**

t1 **=** matrix**[**0**]**

t2 **=** matrix**[**2**]**

v1 **=** matrix**[**1**]**

v2 **=** matrix**[**3**]**

times **=** **[]**

result **=** **[]**

# compare times

**for** indx2**,** i **in** enumerate**(**t2**):**

**for** indx1**,** j **in** enumerate**(**t1**):**

**if** i **==** j**:**

#print "i: %f; j: %f" % (i, j)

times**.**append**(**matrix**[**2**][**indx2**])**

result**.**append**(**v2**[**indx2**]** **-** v1**[**indx1**])**

**return** times**,** result

**def** make_csv**(**filename**,** times**,** delta**):**

**with** open**(**filename**,** 'wb'**)** **as** csvfile**:**

spamwriter **=** csv**.**writer**(**csvfile**,** delimiter**=**';'**,** quotechar**=**'|'**,** quoting**=**csv**.**QUOTE_MINIMAL**)**

spamwriter**.**writerow**([**"time (s)"**,** "residuals (nm)"**])**

**for** i **in** xrange**(**len**(**times**)):**

xstr **=** "%.1f" **%** **(**times**[**i**])**

xstr **=** xstr**.**replace**(**','**,** '.'**)**

ystr **=** "%.4f" **%** **(**delta**[**i**])**

ystr **=** ystr**.**replace**(**','**,** '.'**)**

spamwriter**.**writerow**([**xstr**,** ystr**])**

h**,**c **=** open_csv**(**"foo.csv"**)**

nr **=** nr_of_datasets**(**h**)**

m **=** create_mat**(**c**,**nr**)**

t**,** r **=** calc_diff**(**m**)**

make_csv**(**"bar.csv"**,** t**,** r**)**
